# Surface Replication, Fidelity and Data Loss in Traditional Dental Microwear and Dental Microwear Texture Analysis

**DOI:** 10.1038/s41598-018-37682-5

**Published:** 2019-02-07

**Authors:** Matthew C. Mihlbachler, Melissa Foy, Brian L. Beatty

**Affiliations:** 1 New York Institute of Technology College of Osteopathic Medicine, Old Westbury, NY 11568 USA; 2 Division of Paleontology, American Museum of Natural History, New York, NY 10024 USA

## Abstract

Dental microwear studies often analyze casts rather than original surfaces, although the information loss associated with reproduction is rarely considered. To investigate the sensitivity of high magnification (150x) microwear analysis to common surface replication materials and methods, we compared areal surface texture parameters (ISO 25178-2) and traditional microwear variables (pits and scratches) generated from teeth and casts of rat molars exposed to experimental diets involving hard and soft foods in which abrasive materials had been added. Although the data from the original and replicated surfaces were correlated, many significant differences were found between the resulting data of the casts and original teeth. Both areal surface texture parameters and traditional microwear variables showed diminished ability to discriminate between the eight diet treatments when casts were analyzed. When areal surface texture parameters and traditional microwear variables were combined into a single discriminant function analysis, the cast data and original data produced the most similar results. Microwear researchers tend to favor either texture analysis or traditional microwear methods, better results may be generated by combining them. Although surface textures were not accurately reproduced by the casts, they retained sufficient information to discriminate between microwear of the experimental diets to a degree similar to the original teeth.

## Introduction

Dental microwear analysis is used to test hypotheses about diets and feeding behaviors of ancient vertebrates^1,2^. Most dental microwear studies are based on replications of tooth surfaces, generally made from clear epoxy casts taken from polyvinylsiloxane impressions (molds) of tooth surfaces. Replications are widely used for pragmatic reasons. For example, skulls and mandibles are too large to fit under a microscope. It is comparatively easy to create a series of smaller and more manageable molds and casts. Replications are inevitably imperfect and will result in changes to the surface on some scale. Several studies evaluate impression materials for replicating dental surfaces^3–6^ and other kinds of surfaces^7–12^. However, few studies examine the surface impression materials in the context of dental microwear^13–15^. While the potential for error in dental microwear analysis has been widely recognized^16–20^, most dental microwear studies make no mention of the potential for error due to the replication materials used.

Despite the inevitability of information loss, researchers abundantly find ecologically correlated patterns in microwear data derived from epoxy casts of the teeth of extant species. It is therefore obvious that replications retain valuable information, but we do not yet understand the extent of information loss, or the sensitivities of different methods, magnifications, and resolutions to those sources of error.

**Figure 1:**
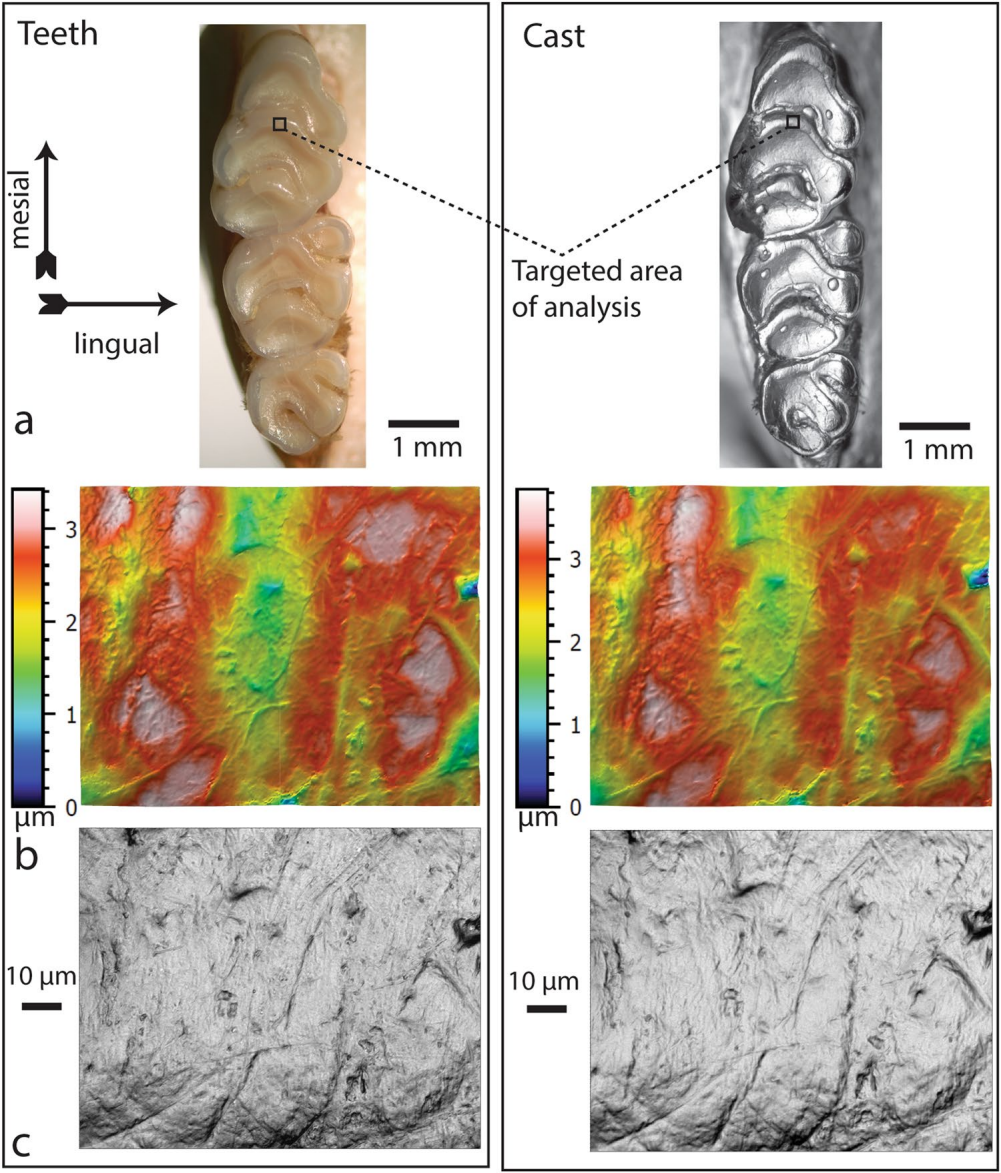
(**a**) Area of analysis on right upper M1s of original teeth and casts; (**b**) color topography map with color Z scale; (**c**) grayscale photosimulation of the same surface.

In this paper, we compare the dental microwear of original tooth surfaces and clear epoxy casts made from polyvinlysiloxane impression material (Fig. 1). Prior studies attempted to compare epoxy casts generated from multiple impression materials including the Colténe Whaledent President Jet Product line, a product commonly used to generate molds for dental microwear research. Based on visual inspection of SEM micrographs, Galbany *et al*.^13,14^ concluded low and mid viscosity polyvinylsiloxane impression media produced the highest fidelity replications. A comparison of seven types of silicone-based impression media to real tooth surfaces using areal surface texture analysis (ISO 25178-2 and SSFA) resulted in similar conclusions^15^. The positive casting media used in these studies was not investigated and included an unspecified polyurethane^14^, and epoxy resins, Epotek 301^13^ and Epotek 320LV^15^. In all three of these studies, Colténe Whaledent President Jet Regular Body, an impression medium frequently used in dental microwear research, was the best or among the best performing among the tested impression media.

Here we attempt to provide additional analysis of tooth replications made from Colténe Whaledent President Jet Regular Body impression material (for molds) and Epokwick Epoxy Resin (20-8136-128) and hardener (20-20-8138-032) (for casts) using upper first molars (M1) of *Rattus norvegicus* that were divided into eight treatments based on different experimental diets (Mihlbachler *et al*. in review). Dental casts were compared to the original tooth surfaces using both a traditional dental microwear method (TM) and dental microwear texture analysis (DMTA) using international standards relating to the analysis of 3D areal surface texture (ISO 25178-2).

At some scale, microwear features on surface replications will have softened edges and rounder peaks and valleys due to the viscosity of the molding and casting compounds. The effects that these changes have on resulting microwear data may depend on the method of microwear analysis. TM involves a human observer who counts discrete abrasion scars (microwear features) and groups them according to size and shape^21–26^. Most prior concerns about TM involve its proneness to high rates of observer error^16–18^. However, TM studies use low magnification and/or low digital resolutions^18,22^ and although individual microwear features may loose some amount of depth and clarity due to viscosity of molding and casting compounds, they will retain their overall sizes, shapes, positions and orientations and are therefore likely to be categorized in the same way on casts (as scratches and pits) as they would be on the original specimens. We hypothesize that TM data will be minimally effected on reproduced surfaces than DMTA because the categorization of abrasion scars according to size and shape (as scratches and pits) does not rely on absolute relief or angularity of surface edges.

**Figure 2:**
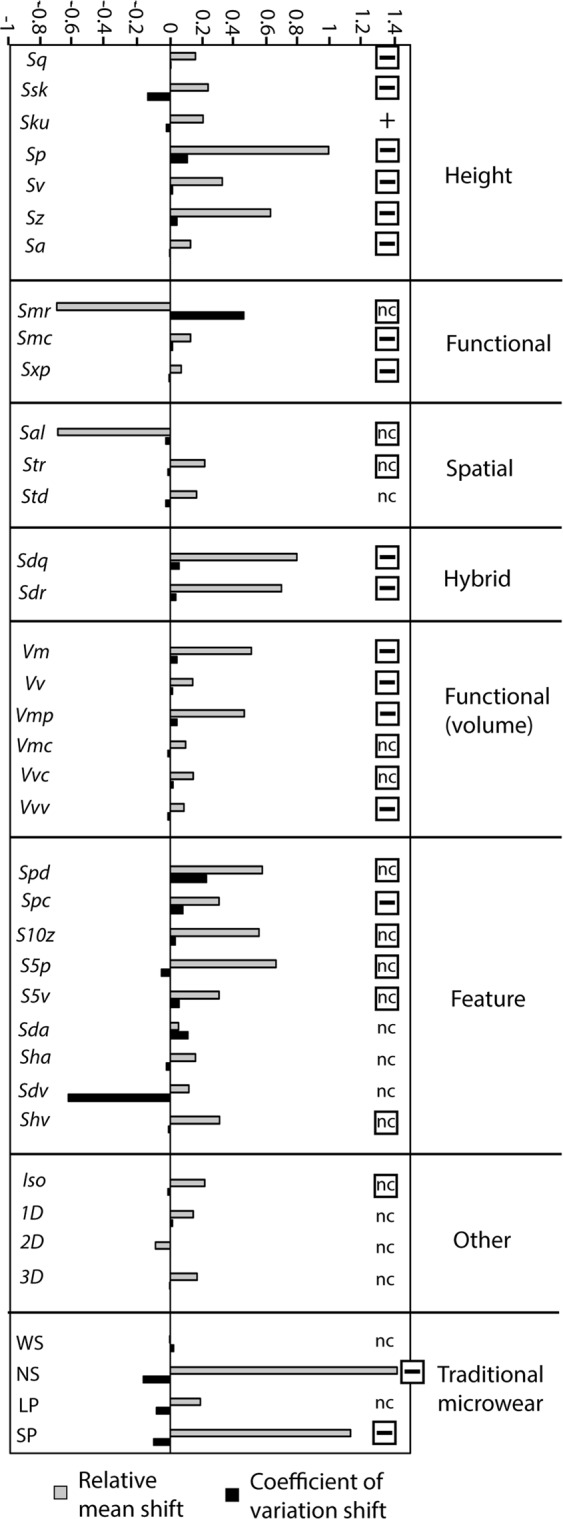
(a) Relative mean shifts (grey bars) normalized for the magnitude of the standard deviation of the original data and coefficient of variation shifts (black bars) of ISO parameters in the dental casts compared to the original surfaces. Positive values are instances where the casts generate greater values (either in the mean or standard deviation) and negative values are instances where casts of surfaces generate lesser values. The symbols to the right are predictions of whether the values produced by the casts should be greater (+), lesser (−) or unchanged (nc) from the values produced by the original surfaces. These symbols are enclosed in a square when the findings did not meet expectations, according to both the polarity (either positive or negative) and the significance of the mean shift (based on paired-T test results shown in Table 1). See Table 1 for abbreviations.

Dental microwear texture analysis (DMTA) involves quantitative analysis of surface textures using confocal and/or focus variation microscopy^27–32^. DMTA largely eliminates human subjectivity but has generated new concerns about instrument inconsistency^33^. Additionally, DMTA more wholly measures surface texture and at finer scales than TM and error associated with replication could be a more significant problem than for traditional microwear (TM). Surface texture variables that are sensitive to the degree of relief and angularity of indentation features seem most likely to be sensitive to surface distortions due to replication. Aspects of surface texture strongly effected by surface replication could include the depths of microwear features, the sharpness of their edges, and the slopes of their walls. On the other hand, surface replications are expected to more accurately preserve other aspects of surface texture such as those related to the orientations, sizes, and overall shapes of indentation scars, and distances between indentations. We hypothesize that ISO texture parameters most strongly associated with relief and angularity will be most strongly affected while parameters more closely related to orientation, size, overall shapes of indentation scars, and distances between them will be less affected (Fig. 2).

Generally speaking, for both TM and DMTA, if cast surfaces are significantly homogenized compare to the original specimens, microwear analysis based on data collected from the casts should produce fewer significant differences between the feeding treatments than analysis of the original teeth. We refer to this type of erroneous result as type II “replication error” because they produce the same type of erroneous conclusion as a classic type II statistical error where a null hypothesis (of undifferentiated microwear) is erroneously accepted. Because information loss should lead to greater homogenization of microwear, the opposite type of error, where significantly different microwear textures emerge on the casts when none exist on the original specimens, is much less likely to occur. This type of error can be called a type I “replication error” due to its analogous relationship to type I statistical error where the null hypothesis (of undifferentiated microwear) is erroneously rejected. We hypothesize that if the surfaces on the casts are significantly homogenized, type II replication error will be the dominant type of disagreement between analysis of the casts and original teeth. Finally, if aspects of dental microwear are significantly degraded, the replications will be less successful to some degree than the original surfaces in correct post hoc classifications of the specimens to their respective feeding treatments based on discriminant function analyses.

## Results

### Combined group results

Descriptive statistics for the ISO and TM variables are in Supplementary Tables 1 and 2. Significant correlations (P > 0.001) between original and cast data were found in all variables, except one, ISO parameter *2D* (P = 0.342). For ISO parameters, Pearson correlation coefficients (PCC) are as high as 0.919 (*Sq*) with an average PCC of 0.66 (Table 1). The narrow scratch (NS) data are the most highly correlated among the TM variables (PCC = 0.455) (Table 2) and the average PCC for TM variables is 0.410.

**Table 1:** ISO surface metrology parameters and statistical results for tooth and cast surfaces.

parameter name	explanation	units	PCC	t statistic (P)	F statistic (P) for teeth	F statistic (P) for casts	Refs
**height parameters**							
*Sq*	Root-mean-square-height	μm	**0**.**919**	**4**.**18 (< 0**.**001)**	**4**.**31 (<0**.**001)**	**3**.**95 (<0**.**001)**	^7^
*Ssk*	Skewness		**0**.**492**	**2**.**58 (0**.**011)**	1.60 (0.142)	1.76 (0.088)	^3^
*Sku*	Kurtosis		**0**.**721**	**2**.**88 (0**.**005)**	**2**.**30 (0**.**031)**	1.36 (0.160)	^1^
*Sp*	Maximum peak height		**0**.**653**	**8**.**01 (<0**.**001)**	**2**.**68 (0**.**013)**	**1**.**99 (0**.**002)**	^4^
*Sv*	Maximum pit height	μm	**0**.**817**	**5**.**12 (<0**.**001)**	**3**.**00 (0**.**006)**	**3**.**78 (0**.**005)**	^3^
*Sz*	Maximum height	μm	**0**.**821**	**8**.**71 (<0**.**001)**	**3**.**08 (0**.**005)**	**3**.**19 (0**.**002)**	^3^
*Sa*	Arithmetic mean height	μm	**0**.**918**	**3**.**48 (0**.**001)**	**4**.**56 (<0**.**001)**	**4**.**13 (<0**.**001)**	^5^
**functional parameters**							
*Smr*	Areal material ratio	%	**0**.**462**	**−8**.**09 (<0**.**001)**	**2**.**69 (0**.**013)**	**1**.**51 (0**.**026)**	^1^
*Smc*	Inverse areal material ratio	μm	**0**.**917**	**3**.**37 (0**.**001)**	**4**.**60 (<0**.**001)**	**3**.**54 (<0**.**001)**	^2^
*Sxp*	Extreme peak height	μm	**0**.**899**	1.75 (0.082)	**3**.**46 (0**.**002)**	**4**.**36 (0**.**004)**	^3^
**spatial parameters**							
*Sal*	Autocorrelation length	μm	**0**.**697**	**−10**.**74 (<0**.**001)**	1.53 (0.163)	1.32 (0.330)	^2^
*Str*	Texture-aspect ratio		**0**.**635**	**2**.**67 (0**.**009)**	1.03 (0.417)	0.51 (0.842)	^3^
*Std*	Texture direction		**0**.**526**	1.97 (0.051)	0.86 (0.543)	1.34 (0.319)	^2^
**hybrid parameters**							
*Sdq*	Root-mean-square gradient		**0**.**730**	**8**.**51 (<0**.**001)**	**7**.**58 (<0**.**001)**	**8**.**18 (<0**.**001)**	^4^
*Sdr*	Developed interfacial area ratio		**0**.**732**	**7**.**73 (<0**.**001)**	**7**.**12 (<0**.**001)**	**7**.**39 (<0**.**001)**	^4^
**functional parameters (volume)**							
*Vm*	Material volume	μm^3^/μm^2^	**0**.**692**	**5**.**74 (<0**.**001)**	**2**.**55 (0**.**018)**	**1**.**42 (0**.**031)**	^3^
*Vv*	Void volume	μm^3^/μm^2^	**0**.**918**	**3**.**67 (<0**.**001)**	**4**.**59 (<0**.**001)**	**3**.**52 (<0**.**001)**	^3^
*Vmp*	Peak material volume	μm^3^/μm^2^	**0**.**590**	**4**.**65 (<0**.**001)**	**3**.**13 (0**.**005)**	**1**.**42 (0**.**009)**	^4^
*Vmc*	Core material volume	μm^3^/μm^2^	**0**.**903**	**2**.**55 (0**.**012)**	**4**.**72 (<0**.**001)**	**4**.**33 (<0**.**001)**	^6^
*Vvc*	Core void volume	μm^3^/μm^2^	**0**.**913**	**3**.**64 (<0**.**001)**	**4**.**62 (<0**.**001)**	**3**.**37 (<0**.**001)**	^6^
*Vvv*	Pit void volume	μm^3^/μm^2^	**0**.**887**	**−28**.**50 (<0**.**001)**	**2**.**64 (0**.**015)**	**4**.**16 (0**.**018)**	^7^
**feature parameters**							
*Spd*	Density of peaks	1/μm^2^	**0**.**469**	**3**.**87 (<0**.**001)**	**2**.**79 (0**.**010)**	**0**.**76 (0**.**008)**	^5^
*Spc*	Arithmetic mean peak curvature	1/μm	**0**.**709**	**3**.**41 (0**.**001)**	**6**.**51 (<0**.**001)**	**4**.**79 (<0**.**001)**	^2^
*S10z*	Ten point height	μm	**0**.**763**	**7**.**40 (<0**.**001)**	**3**.**25 (0**.**003)**	**3**.**51 (0**.**003)**	^1^
*S5p*	Five point peak height	μm	**0**.**497**	**6**.**96 (<0**.**001)**	**3**.**29 (0**.**003)**	**1**.**17 (0**.**001)**	^1^
*S5v*	Five point pit height	μm	**0**.**697**	**3**.**66 (<0**.**001)**	**2**.**75 (0**.**011)**	**4**.**64 (0**.**005)**	^4^
*Sda*	Mean dale area	μm^2^	**0**.**211**	0.44 (0.663)	0.98 (0.453)	1.38 (0.265)	^2^
*Sha*	Mean hill area	μm^2^	**0**.**487**	1.69 (0.093)	**2**.**11 (0**.**048)**	1.45 (0.089)	^2^
*Sdv*	Mean dale volume	μm^3^	**0**.**194**	1.13 (0.260)	0.79 (0.594)	1.61 (0.101)	^4^
*Shv*	Mean hill volume	μm^3^	**0**.**377**	**2**.**64 (0**.**009)**	1.56 (0.155)	1.24 (0.395)	^3^
**Other**							
*Iso*	Isotropy		**0**.**635**	**2**.**67 (0**.**009)**	1.03 (0.417)	0.51 (0.842)	
*1D*	First Direction		=**0**.**615**	1.70 (0.091)	0.74 (0.639)	0.76 (0.373)	
*2D*	Second Direction		0.086	0.75 (0.456)	0.13 (0.996)	0.70 (0.998)	
*3D*	Third Direction		=**0**.**319**	1.58 (0.118)	1.02 (0.418)	0.71 (0.359)	

PCC (Pearson Correlation Coefficients) and reported t statistics (and p values) for related samples paired T tests are results that compare teeth to casts with all feeding treatments combined. F statistics (and P values) of ANOVAs independently test the ability of teeth and casts to find differences between the feeding treatments. All tests have 122 degrees of freedom. Bold results are significant (P ≤ 0.05). Column Refs. indicates the number of references, out of ten, in which significant dental microwear wear differences were found for each ISO surface metrology parameter^27,30,35,40–46^.

**Table 2:** Traditional microwear variables and statistical results of tooth and cast surfaces.

variable name	definition	PCC	t statistic (P)	F statistic (P) for teeth	F statistic (P) for casts
wide scratches (WS)	max width = 1.25–2.5 μm	**0**.**435**	0.04 (0.967)	0.66 (0.707)	1.79 (0.631)
narrow scratches (NS)	max width > 2.5 μm	**0**.**455**	**−6**.**89 (<0**.**001)**	**9**.**91 (<0**.**001)**	**3**.**38 (<0**.**001)**
large pits (LP)	max diameter 2.5–5 μm	**0**.**411**	−1.96 (0.052)	**3**.**30 (0**.**003)**	**5**.**29 (<0**.**001)**
small pits (SP)	max diameter > 2.5 μm	**0**.**340**	**10**.**85 (<0**.**001)**	**4**.**00 (0**.**001)**	**3**.**06 (<0**.**001)**

PCC (Pearson Correlation Coefficients) and Paired T (Related samples paired T tests) are results that compare teeth to casts with all feeding treatments combined. ANOVA independently tests the ability of teeth and casts to find differences between the feeding treatments. All tests have 119 degrees of freedom. Bold results are significant (P ≤ 0.05).

With the feeding groups combined, most (26 of 34) ISO parameters differed significantly between original and cast surfaces according to paired T-tests (Table 1). On average, the absolute value of the relative mean shift was 0.24 (Fig. 2). *Sp*, the most strongly effected parameter, had a relative mean shift of 0.99, indicating the mean of the cast data was shifted from the original data to a degree that is nearly equal in magnitude to the standard deviation of the original data. In most cases, the changes to the mean values associated with analysis of casts were positive with higher values. A strong mean shift was negative in only a small number of cases (*Smr*, *Sal*).

For TM variables, significant differences between original and cast surfaces were found for NS (narrow scratches) and SP (small pits) (Table 2). The relative mean shifts for NS (1.42) and SP (1.13) were the highest encountered in this study. The relative mean shifts of these variables were highly positive.

Although casting altered the absolute magnitude of the values for ISO parameters and TM variables, it did not lead to changes in the amount of variation in the data. The coefficient of variation shift was small on average (0.07) and only two ISO parameters had unusually high changes in coefficients of variation in either positive (*Smr*) or negative directions (*Sdv*). Differences in coefficients of variation among the TM series of variables were similarly small (Fig. 2).

### ANOVA

ANOVAs tested for differences in microwear between the feeding treatments. ANOVA of the original data found significant differences between feeding trials in 23 out of 34 ISO parameters (Table 1) and in three of four TM variables (Table 2). ANOVA of the cast data found an identical set of significant results with the exceptions of two ISO parameters (*Sha* and *Sku*) for which the significant results in the original data were not replicated with the cast data (type II replication errors).

In Tukey’s Post hoc pairwise comparisons made of the pellet-fed control group to the remaining treatments, the original data found 18 significant pairwise differences among the ISO parameters involving feeding treatments Pde, Dcc, Dde, and Dqs (Table 3). The cast data failed to find half (9) of these differences (type II replication errors) and only produced significant results involving one treatment (Dqs). One type I replication error, a falsely significant result, was produced where ISO parameter *Sdq* was found to significantly differ between treatments P and Dqs in the cast data but not in the original data.

**Table 3:** Tukey’s test P-values for pairwise comparisons of ISO parameters for which significant differences were found between the pellet diet control group and other diet groups for tooth surfaces.

Pellet vs	*Sq*	*Sku*	*Sa*	*Smc*	*Sxp*	*Sdq*	*Sdr*	*Vm*	*Vv*	*Vmp*	*Vmc*	*Vvc*	*Vvv*	*Spd*	*S10z*	*S5p*
**Tooth surfaces**																
D	0.769	0.332	0.677	0.704	0.837	1.000	1.000	0.999	0.717	0.999	0.531	0.721	0.921	0.664	0.996	0.866
Pcc	0.706	0.443	0.538	0.536	0.974	0.999	1.000	0.998	0.553	0.998	0.402	0.497	0.997	0.953	0.632	0.889
Pde	1.000	**0**.**026**	0.999	0.996	1.000	**0**.**021**	0.096	1.000	0.996	1.000	0.985	0.992	1.000	0.479	0.999	1.000
Pqs	0.803	0.150	0.685	0.805	0.792	1.000	1.000	1.000	0.820	0.999	0.532	0.835	0.902	0.976	1.000	0.975
Dcc	0.695	0.450	0.642	0.743	0.817	0.996	1.000	0.763	0.734	0.768	0.540	0.758	0.818	**0**.**041**	0.989	0.995
Dde	0.869	0.053	0.756	0.695	0.994	0.072	0.207	0.916	0.694	0.918	0.666	0.622	1.000	**0**.**040**	1.000	0.958
Dqs	**0**.**000**	**0**.**029**	**0**.**000**	**0**.**000**	**0**.**002**	0.190	**0**.**045**	**0**.**028**	**0**.**000**	**0**.**006**	**0**.**000**	**0**.**000**	**0**.**025**	0.177	**0**.**016**	**0**.**014**
**Cast surfaces**																
D	0.760	0.981	0.738	0.908	0.571	0.997	0.967	0.995	0.910	0.995	0.585	0.938	0.580	0.999	1.000	1.000
Pcc	0.940	1.000	0.891	0.936	0.997	0.982	0.813	1.000	0.942	1.000	0.770	0.937	0.996	1.000	1.000	1.000
Pde	0.997	0.544	1.000	1.000	0.979	0.109	0.348	0.999	1.000	0.999	1.000	1.000	0.908	0.975	0.615	0.989
Pqs	0.990	0.899	0.969	0.999	0.951	1.000	0.999	1.000	0.999	1.000	0.876	1.000	0.977	0.999	1.000	1.000
Dcc	0.700	1.000	0.643	0.897	0.541	0.998	0.966	0.979	0.896	0.979	0.519	0.925	0.595	0.880	0.988	1.000
Dde	0.992	0.414	0.971	0.996	1.000	0.838	0.983	0.990	0.996	0.990	0.907	0.993	1.000	0.810	0.991	0.993
Dqs	**0**.**003**	0.500	**0**.**001**	**0**.**003**	**0**.**005**	**0**.**002**	**0**.**044**	0.377	**0**.**004**	0.377	**0**.**000**	**0**.**004**	**0**.**020**	1.000	0.162	0.703

The value 0.000 indicates P < 0.001, all other values are equal to P as reported by SPSS. Bold results are significant (P ≤ 0.05). D = dough; Dcc = Dough w/calcium carbonate; Dde = Dough w/diatomaceous earth; Dqs = Dough w/quartz sand; P = pellet; Pcc – Pellet w/calcium carbonate; Pde = Pellet with diatomaceous earth; Pqs = Pellet w/quartz sand. See Table 1 for ISO parameter abbreviations. ISO parameters with no significant results are not shown.

For TM variables, Tukey’s Post hoc pairwise comparisons of the control (P) with the other diet treatments found four significant differences. The cast data failed to replicate two of these results (type II replication errors) and produced one erroneously significant difference (type replication I error) (Table 4).

**Table 4:** Tukey’s test P-values for pairwise comparisons of TM variables between the pellet diet control group and other diet groups for tooth surfaces.

Pellet vs	WS	NS	LP	SP
**Tooth surfaces**				
D	1.000	**0**.**009**	0.995	0.998
Pcc	0.997	1.000	1.000	1.000
Pde	0.999	**0**.**010**	1.000	0.295
Pqs	0.951	0.576	0.999	1.000
Dcc	1.000	0.392	1.000	0.953
Dde	0.995	0.108	**0**.**032**	0.073
Dqs	1.000	**0**.**000**	1.000	0.973
**Cast surfaces**				
D	1.000	0.989	1.000	1.000
Pcc	0.353	0.969	0.988	0.968
Pde	0.983	0.783	**0**.**035**	0.135
Pqs	1.000	1.000	1.000	0.991
Dcc	1.000	1.000	0.273	0.429
Dde	1.000	0.905	**0**.**020**	0.402
Dqs	1.000	**0**.**028**	0.971	0.999

Bold results are significant (P ≤ 0.05). See Table 1 for TM variable abbreviations and Table 5 for feeding group abbreviations.

### Discriminant Function Analysis

**Figure 3:**
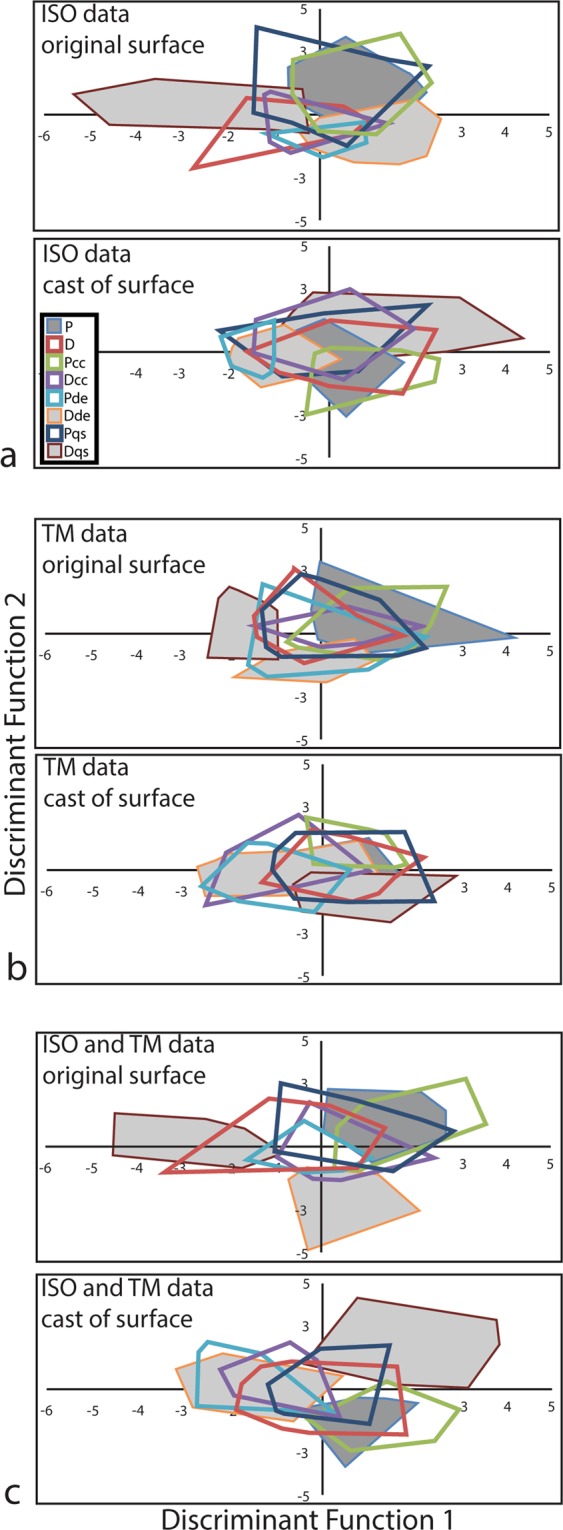
Outlines of areas occupied by the eight feeding treatments on discriminant functions one (x-axis) and two (y-axis) of original tooth surfaces and casts of ISO data, TM data, and total evidence. See Table 3 for diet group abbreviations.

Comparisons of the DFA of the original and cast data reveal the abilities of casts to preserve discriminatory aspects of surface texture. All DFAs were significant (P < 0.001) (Table 5), however, for both TM and ISO, casts were 9.2 and 10.6 percentage points less successful than original data at post hoc classifications of specimens to their respective feeding treatments resulting in more group overlap in plots of the first and second discriminant functions for casts (Fig. 3a,b). The total evidence DFA performed better overall with the highest rates of correct post hoc classification, and the cast data produced a rate of correct rate of post hoc classification (68.9%) that is only 3.4 percentage points less than that of the original data (72.3%) (Table 5, Fig. 3c).

**Table 5:** Results of discriminant function analyses on data from original tooth surfaces and cast surfaces.

	Chi-Squared	Sig. (P)	Wilks’ Lambda	% correctly classified
**Tooth surfaces**				
TM	103.562	P ≤ 0.001	0.400	41.7
ISO	274.215	P ≤ 0.001	0.073	60.2
ISO + TM	319.873	P ≤ 0.001	0.039	72.3
**Cast Surfaces**				
TM	80.593	P ≤ 0.001	0.490	32.5
ISO	161.824	P = 0.006	0.228	49.6
ISO + TM	299.853	P ≤ 0.001	0.050	68.9

Canonical loadings (Supplementary Tables S8 and S9) are the correlations of the individual variables with the discriminant function. Ideally, perfect replications would produce a canonical structure identical to the original data, however the resulting canonical structures of the original and cast data are different. There is only a low degree of correlation between the canonical loadings of the first DFs of cast and original data (PCC = −0.443; P = 0.005) and there is no significant correlation between the second DFs (PCC = 0.034; P = 0.839). The canonical structures of the two datasets appear to be inverted in the sense that the first DF of the cast data is much more highly correlated to the second DF of the original data (PCC = 0.922: P < 0.001), and vice versa (PCC = 0.774; P < 0.001), therefore, similar canonical axes were produced by these datasets, albeit in different orders of significance (Supplementary Fig. S1).

**Figure 4:**
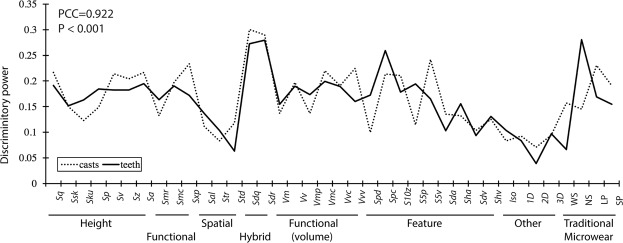
Total discriminatory power of ISO parameters and TM variables as a function of the scaled sums of the canonical loadings of all 7 discriminant functions based on the total evidence analysis. See Tables 1 and 2 for abbreviations.

Although the above results indicate the canonical structure has been distorted, the discriminatory power of each ISO and TM variable was largely preserved. Calculations of the total discriminatory power of each ISO parameter and TM variable produced correlated results between original surfaces and casts (Pearson Correlation coefficient = 0.922; P < 0.001) (Fig. 4). In both sets of analyses, the hybrid parameters (*Sdq* and *Sdr*) had the highest overall discriminatory power among the ISO parameters. Among the TM variables, narrow scratches (NS) had the highest discriminatory power in the original data, but the influence of this variable was reduced in the cast data.

## Discussion

Research questions that are concerned with understanding contact mechanics and wear may require accurate measurement of surface texture. The casts produced data that were significantly different from the original surfaces. Goodall *et al*.^15^ found very few significant differences between tooth surfaces and replicated surfaces based on the same Colténe Whaledent President Jet Regular Body impression material. The discrepancy between our results (many differences) and those of Goodall *et al*.^15^ (few differences) may be related to the specific type of surface textures studied, poor quality control of the impression material itself, different epoxy resins, instrumentation differences, or differences in magnification. The Goodall *et al*.^15^ study was performed at a lower magnification (100x) than our study (150x). It is likely that magnification had some effect on these different results if the scale of the imperfections in the replications was similar in the two studies. Some researchers analyze the negative impressions^34,35^, which, when digitally scanned, can readily be inverted into the positive surface. Analysis of molds removes one step from the reproduction process and future tests on the efficacy of molds will review where and to what degree information is lost in the replication process.

Most dental microwear analyses test hypotheses by associating different microwear patterns with different diets or other aspects of feeding ecology. This objective doesn’t require accurate replication of true surface textures as long as unique aspects of texture are maintained in the replications. In this study, ANOVAs of both original and cast data found similar sets of ISO parameters and identical sets of TM variables for which significant differences between the diets occurred (Tables 1 and 2). However, casts performed poorly in the posthoc pairwise comparisons of the control (P) to the remaining feeding groups and found fewer significant pairwise differences than the original surfaces (Tables 3 and 4). We correctly hypothesized that cast data would produce more instances of type II replication error where microwear differences found on the original surfaces would not be found on the casts. Therefore, there is evidence for considerable information loss in the casts.

**Figure 5:**
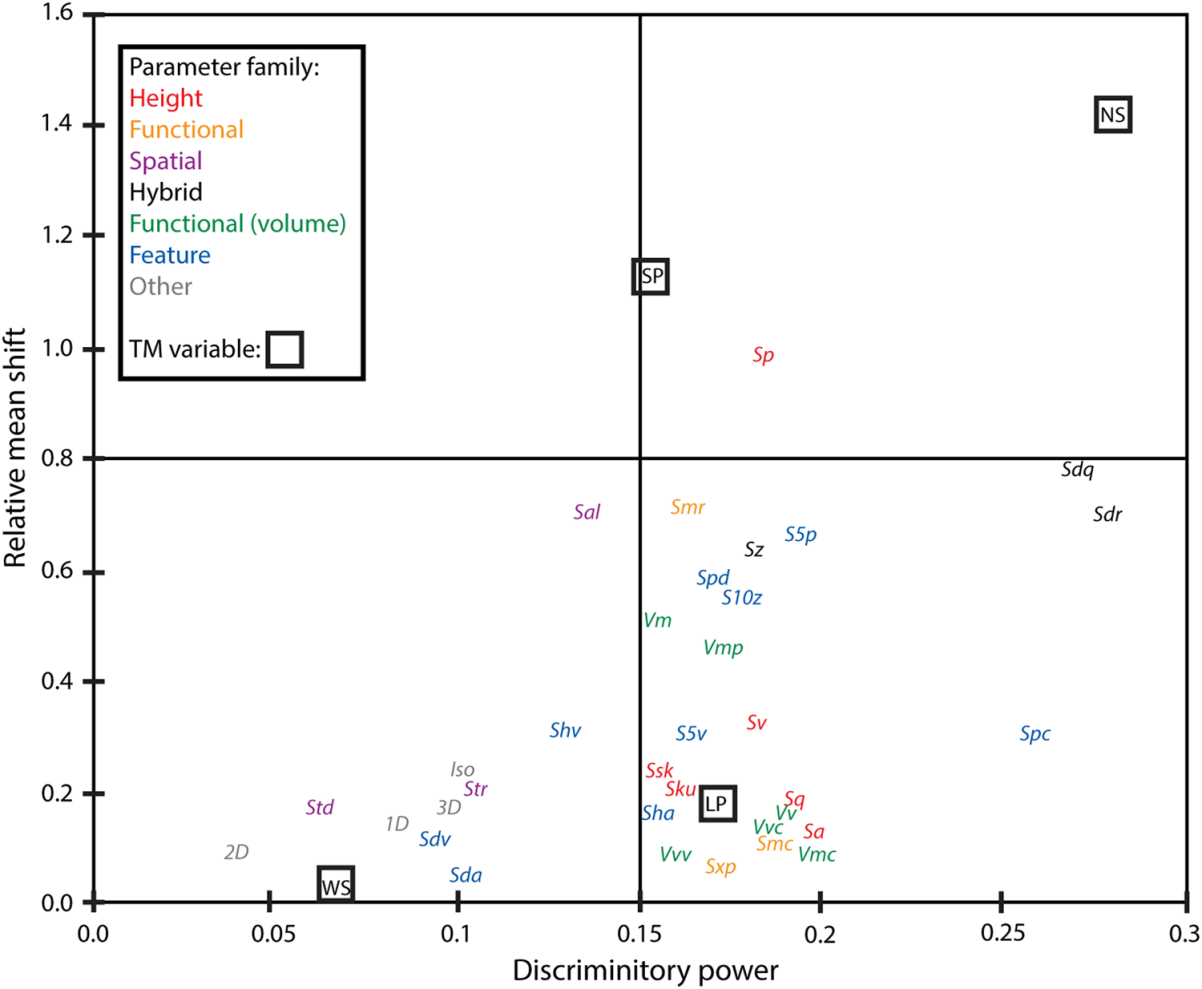
Discriminatory power (x-axis) versus relative mean shift (Y axis) of ISO parameters and TM variables. Variables appearing in the lower right quadrant have both ideal characteristics: low sensitivity to replication and high discriminatory power. See Tables 1 and 2 for abbreviations.

We surveyed published microwear studies that use ISO 25178-2 (Table 1). The five parameters found to most frequently produce significant findings in the literature survey (*Sq*, *Sa*, *Vmc*, *Vvc* and *Vvv*) also performed very well in our comparison of original surfaces and replications. The means and standard deviations of these five parameters were not strongly altered in the cast data. Significant differences in these parameters were found in both the original and cast data and they produced relatively high discriminatory power (Fig. 5). The two hybrid parameters, *Sdq* and *Sdr*, are examples of parameters that were not accurately replicated in the casts but nonetheless were the most discriminating ISO parameters in both the casts and original dental surfaces (Fig. 5).

Although the retention of discriminatory power in the replications is promising for dental microwear research, other inexplicable results emerged from the comparison of original and cast data. Figure 2 includes predictions for change for each parameter in the average value across the total sample for each variable based on definitions of ISO parameters^36^ and on the overall hypothesis that microwear features on cast surfaces should have lower relief, less angularity, and as a result, less clarity than the original features. Those features related to relief and angularity we predicted to undergo decreases in average values. For other parameters we predicted no (or minimal) change. Perplexingly the casts produced a large number of unusual results primarily in the form of higher values of surface texture parameters and TM variables.

It is possible that additional variables, such as the differential reflectivity of enamel and the casting material (clear epoxy) were additional variables that influenced the results^11^. In light microscopy, clear epoxy casts are preferable to the original teeth due to their greater reflectance. Sputter coating may enhance reflectance. We suggest sputter coating both original specimens and epoxy casts would be a means of standardizing the reflectance of the surfaces and may offer a more controlled means of measuring the texture differences between original dental specimens and surface casts.

The hypothesis that TM is robust to replication is not supported. TM analysis occurs at the scale of individual microwear features. While the faintest of microwear features might be lost in the casts, changes to the depths and sharpness of microwear features have little bearing on their classification as pits or scratches. However, TM was found to be scale sensitive to replication. While large microwear features, WS (wide scratches) and LP (large pits), were not significantly affected, narrow scratches and small pits were the most altered variables in the entire study. Inexplicably, significantly higher numbers of small pits and narrow scratches were identified in the casts. The proportionality of pits and scratches was also distorted. The average ratio of total numbers of pits to total numbers of scratches in the original data (3.59) is greater than that of the cast data (3.08). Bivariate plots of pits and scratches from the cast data produce a similar, albeit displaced and somewhat distorted scatter pattern when compared to the original (Supplementary Fig. S2). It is noteworthy that NS had the highest discriminatory value in the total evidence DFA on original teeth, and this variable clearly has importance in dental microwear research for discriminating major dietary categories^37^. However, NS was also the most sensitive to replication, as the discriminatory power of NS was the most strongly diminished variable in the entire study (Fig. 5).

The results reported here demonstrate that the accurate characterization of specific microwear textures on worn tooth surfaces is significantly diminished on casts manufactured from molding and casting materials commonly used by dental microwear researchers. However, if one’s research objective is primarily to discriminate strongly differentiated textures rather than accurate measurement of texture, surface replications seem to be reasonably efficacious methodology. However, the weaker nature of the cast results suggests that subtle differences in microwear texture may be lost in replicates. Although the data produced by the replications seemed adequate for discriminating different microwear textures, their performance was most similar to the original surfaces when ISO and TM data were combined. Microwear researchers tend to favor one method over another, but they may generate better results by combining them.

## Materials and Methods

### Experimental animals and specimen preparation

All methods were performed in accordance with the relevant guidelines and regulations of the New York Institute of Technology Institutional Animal Care and Use Committee following the approved protocol 2014-BB-01. This study utilized the teeth of 138 rats fed experimental diets (Mihlbachler *et al*., in review). Controlled feeding experiments were conducted at NYIT-College of Osteopathic Medicine. The experimental rats were divided into eight varying diet categories. Rats were first designated to either a chow (pellet form) diet or bacon dough (soft form) diet. Within each food type, rat diets were further subdivided into four groups: (1) no added abrasives, (2) calcium carbonate, (3) diatomaceous earth, and (4) quartz sand. Rats were exposed to these special diets for 15 days and were then sacrificed. The control treatment was one in which rats were continued on the same pellet diet with no added abrasives that they had been fed prior to the experiment.

### Replication methods

Following extraction and cleaning (Mihlbachler *et al*. review), Colténe Whaledent President Jet Regular Body was applied to the occlusal surfaces of the right upper molar rows using the applicator gun. The impression materials were allowed a minimum time of 45 minutes to harden. The molds were trimmed with a #22 scalpel blade to remove extraneous material. For purposes of pouring liquid epoxy with the molded dental surfaces facing up, circular retaining walls were hand sculpted around each trimmed mold with Colténe Lab-Putty, a product designed specifically to bond with the impression material. Epokwick Epoxy Resin (20-8136-128) and hardener (20-20-8138-032) were mixed (5:1 by weight) according to manufacturer instructions, degassed in a vacuum chamber at −25 < −30 inHg for five minutes, and poured over the molds. After pouring, each specimen was centrifuged for 1 minute with a hand-cranked centrifuge and left to harden without disturbance for 48 hours. The epoxy casts and molds were left together, placed in plastic bags and separated as needed for microscopy.

### Confocal microscopy

TM and DMTA are based on identical sets of 3D surface scans acquired from a Sensofar Plu NEOX optical profiler. We scanned the mesial enamel ridge of each specimen’s second maxillary molar (M2) at 150x (Fig. 1a). All scans were done with the same orientation with respect to the mesiodistal axis of the tooth. All scans were done with white light with a 150x objective (NA = 0.90, WD = 1.50 mm) with step sizes of 0.10 µm. Each initial scan, prior to extraction of a smaller area, was 1360 × 1024 pixels (each pixel is 0.09 µm). ISO parameters were derived with the default threshold settings in place (SMr c = 1 µm under the highest peak; Smc p = 10%; Sxp p = 50% and q = 97.5%; Sal s = 0.2; Str s = 0.2; Std reference angle is 0°; Vm p = 10%; Vv p = 10%; Vmp p = 10% and q = 80%; Vmc p = 10% and q = 80%; Vvc p = 10% and q = 80%; Vvv p = 80%; Spd pruning = 5%; Spc pruning = 5%; S10z pruning = 5%; S5p pruning = 5%; S5v pruning = 5%; Sda pruning = 5%; Sha pruning = 5%; Sdv pruning = 5%; Shv pruning = 5%).

The same areas on both the casts and original teeth were identified and scanned using individual microwear features that could be identified on both teeth and casts as landmarks to frame the same areas (Fig. 1b). Specimens were excluded when the surfaces had visible contaminants after several cleaning attempts. Each scan resulted in a “.plu” file that was loaded into SensoMAP Premium software (version 7.2.7368). An area of 80 × 100 micrometers was extracted, then leveled using the least square planes method. Leveling was accomplished by subtraction to a least squares plane. The curvatures of the surfaces were removed to reduce the effect of the gross contours of the tooth on the data, using a polynomial of degree 3. A 3D view was produced so that it could be manipulated and viewed in various orientations to visualize problem areas of abnormal peaks and valleys. These problem areas were retouched using the “retouch surface points” operator. Missing data points were filled in by a smooth shape calculated from the nearest neighbors. The parameters table and texture direction studies were then obtained (Table 1). Further details on handling of specimens, positioning, scanning protocols, and processing protocols are provided elsewhere.

### TM analysis

The data files described above were converted to 268 × 214 pixel images with a pixel density of 7.18 pixels per μm^2^ (Fig. 1c) in Adobe Photoshop CC using the Bicubic Sharper interpolation method. The grayscale images derived from the confocal data superficially resemble photomicrographs of clear epoxy casts taken under a light microscope, and these images were analyzed with methods derived from light microscopy methods^18,20^. The confocal scans were converted to 1338 pixel x 1070 pixel images covering the same tooth surface areas of 100 μm × 80 μm from which the ISO data were derived. The digital density of the images was reduced by resampling the images in Adobe Photoshop, resulting in 268 × 214 pixels images with a pixel density of 7.18 pixels per μm^2^. Reducing the digital resolution of high-resolution microwear photomicrographs reduces the potential for observer error without significantly diminishing the differences in dental microwear patterns between samples^18^.

Microwear features were assigned to one of four primary categories as defined in Table 3. In addition to these four primary variables, two secondary variables were calculated: TS (total number of scratches) and TP (total number of pits). Microwear features smaller than the above criteria were not counted because observer repeatability for small poorly resolved microwear features was found to be low with similar methods (Mihlbachler and Beatty 2012).

Microwear features were assigned to four categories as defined in Table 2. Smaller microwear features were excluded. Observer blindness was achieved by randomly ordering and assigning arbitrary numbers to the image files. Microwear features were traced directly on the images by superimposing standardized circles (for pits) and lines (for scratches) in Adobe Illustrator software. The images were examined in random order three times by a single observer. The superimosed tracings were saved after each pass, preserving the observer’s interpretation. Multiple passes allowed the images to be more comprehensively sampled for discrete microwear features by eliminating the diminishing effects of observer fatigue. During each pass, additional features that had been missed in earlier passes were identified. By the third pass, very few additional microwear features were recognized (one or two per image) so additional passes were not made.

### Statistical methods

All statistical analyses were performed on SPSS ver. 24. Of the 138 rat specimens, some were excluded from analysis because we could not get good scans for a variety of reasons (e.g. cracked and damaged enamel, specimen damaged during molding). Some additional specimens were rejected from TM because it was difficult for the observer to differentiate large overlapping wear features. Statistical analyses were run only on specimens for which complete data could be obtained for the group of variables in question (ISO or TM). Analyses of DMTA data included 123 specimens. Analyses of TM data included 120 specimens. Discriminant function analyses included only specimens for which complete data had been recovered. Keeping in mind the large number of statistical tests reported above, we caution that the distributions of significant results are more meaningful than the results of individual tests. Here, we report the results of parametric analyses on the raw data. Some of the data had distributions that deviated significantly from normality (Shapiro-Wilk P ≤ 0.05). Therefore, we ran a parallel series of analyses using rank-transformed data and other nonparametric methods to ensure that erroneous conclusions were not made due to assumption violations. These analyses (Supplementary text and Tables S3–S7) were not identical but similar enough to warrant the same conclusions.

#### Combined analyses

With the eight feeding treatments combined, we ran Pearson Correlation coefficients (PCC) and paired-T tests to compare the original and cast data (Table 1 for ISO, Table 2 for TM).

To summarize the degree to which the mean values of the data were shifted in the replicated surfaces, a ratio, termed the “relative mean shift” (RMS) (Fig. 2) was calculated:

$$RMS=\frac{Mr-Mo}{So}$$

This ratio normalizes the magnitude of the individual parameters according to the standard deviation of the original data.

To summarize the degree to which surface replication homogenized the data, the difference between the coefficient of variation of the cast data and the coefficient of variation of the original data was calculated. This value was termed the “coefficient of variation shift” (CVS) (Fig. 2):

$$CVS=\frac{Sr}{Mr}-\frac{So}{Mo}$$

In the above equations, *Mr* is the mean value of the replicated surface data, *Mo* the mean value of the original data, *So* the standard deviation of the original data, and *Sr* the standard deviation of the replicated surface data.

#### Replication of ANOVA results

ANOVAs were run on original data and then again on the cast data. ANOVAS test for differences between the microwear of the eight feeding treatments (Table 3 for ISO, Table 4 for TM). ANOVA is robust to violations of the assumption of normal distribution^38,39^. Levene’s test was used to test for unequal variances within each dataset. For the majority of data, homogeneity of variance between the feeding treatments could not be falsified. Therefore, Tukey’s Post Hoc tests determined which of the numerous two-way comparisons of the analyses of raw data significantly differ. In cases where ISO parameters were found to have unequal variances between the feeding treatments (*Sdr*, *Spd*, *Spc*) we used Dunnett’s T3, which is a more appropriate test for unequal variances. We report only the two-way comparisons between the control diet (pellets) with the other diet treatments (Table 3 for ISO, Table 4 for TM).

#### Replication of discriminant function analysis results

Two sets of discriminant function analysis (DFAs) were run on the original and cast data to determine the success rate of cast data at predicting diet compared to the original data. DFAs were run using the ISO data alone and the TM data alone (Table). Thirdly, a “total evidence” analysis in which ISO and TM data were combined was run. To examine similarities in the canonical structure of original specimens and casts, we calculated Pearson correlation coefficients on the total evidence canonical loadings of the first and second discriminant function axes.

A final calculation summarized the total influence each ISO parameter and TM variable in the discriminant function analyses. The following calculation considers the diminishing amount of variance explained by each subsequent DF, where c = canonical loading, v equals the percentage of variance explained by each DF, and N is the number of discriminant functions (Supplementary Tables S8 and S9).

$$\sum _{i=1}^{N}{c}^{i}{v}^{i}$$

## Supplementary information

supplementary text, tables, figures

raw data

## Data Availability

All data generated during this study are included as a supplemental data file.

## References

[1] Varriale FJ (2016). Dental microwear reveals mammal-like chewing in the neoceratopsian dinosaur Leptoceratops gracilis. PeerJ.

[2] Ungar, P. S. & Berger, L. R. Brief communication: Dental microwear and diet of Homo naledi. *Am*. *J*. *Phys*. *Anthropol*. n/a-n/a, 10.1002/ajpa.23418 (2017). 10.1002/ajpa.23418 29399788

[3] Fiorenza L, Benazzi S, Kullmer O (2009). Morphology, wear and 3D digital surface models: materials and techniques to create high-resolution replicas of teeth. *J Anthr*. Sci.

[4] Rodrigues HG, Merceron G, Viriot L (2009). Dental microwear patterns of extant and extinct Muridae (Rodentia, Mammalia): ecological implications. Naturwissenschaften.

[5] Bello SM, Verveniotou E, Cornish L, Parfitt SA (2011). 3‐dimensional microscope analysis of bone and tooth surface modifications: comparisons of fossil specimens and replicas. Scanning.

[6] Austin RS, Mullen F, Bartlett DW (2015). Surface texture measurement for dental wear applications. Surf. Topogr. Metrol. Prop..

[7] Chee WW, Donovan TE (1992). Polyvinyl siloxane impression materials: a review of properties and techniques. J. Prosthet. Dent..

[8] DeLong R, Pintado MR, Ko C-C, Hodges JS, Douglas WH (2001). Factors influencing optical 3D scanning of vinyl polysiloxane impression materials. J. Prosthodont..

[9] Nilsson L, Ohlsson R (2001). Accuracy of replica materials when measuring engineering surfaces. Int. J. Mach. Tools Manuf..

[10] Chung S, Im Y, Kim H, Jeong H, Dornfeld DA (2003). Evaluation of micro-replication technology using silicone rubber molds and its applications. Int. J. Mach. Tools Manuf..

[11] Rosén, B.-G., Blunt, L. & Thomas, T. R. On *in-vivo* skin topography metrology and replication techniques. In **13**, 325 (IOP Publishing, 2005).

[12] Bai XQ (2013). Study on biomimetic preparation of shell surface microstructure for ship antifouling. Wear.

[13] Galbany J, Martínez LM, Pérez- Pérez A (2004). Tooth replication techniques, SEM imaging and microwear analysis in primates:Methedological obstacles. Anthropologie.

[14] Galbany J (2006). Comparative analysis of dental enamel polyvinylsiloxane impression and polyurethane casting methods for SEM research. Microsc. Res. Tech..

[15] Goodall RH, Darras LP, Purnell MA (2015). Accuracy and precision of silicon based impression media for quantitative areal texture analysis. Sci. Rep..

[16] Grine FE, Ungar PS, Teaford MF (2002). Error rates in dental microwear quantification using scanning electron microscopy. Scanning.

[17] Galbany J (2005). Error rates in buccal-dental microwear Quantification using scanning electron microscopy. Scanning.

[18] Mihlbachler MC, Beatty BL (2012). Magnification and resolution in dental microwear analysis using light microscopy. Palaeontol. Electron..

[19] DeSantis LRG (2013). Direct comparisons of 2D and 3D dental microwear proxies in extand herbivorous and carnivorous mammals. PLoS ONE.

[20] Mihlbachler MC, Beatty BL, Caldera-Siu A, Chan D, Lee R (2012). Error rates in dental microwear analysis using light microscopy. Palaeontol. Electron..

[21] Solounias N, Semprebon G (2002). Advances in reconstruction of ungulate ecomorphology with applications to early fossil equids. Am. Mus. Novit..

[22] Semprebon G, Godfrey L, Solounias N, Sutherland MR, Jungers WL (2004). Can low-magnification stereomicroscopy reveal diet?. J. Hum. Evol..

[23] Solounias N, Rivals F, Semprebon GM (2010). Dietary interpretation and paleoecology of herbivores from Pikermi and Samos (late Miocene of Greece). Paleobiology.

[24] Green, J. L. & Kalthoff, D. C. Xenarthran dental microstructure and dental microwear analysis, with new data for *Megatherium americanum* (Megatheriidae). *J*. *Mammal*. 10.1093/jmamma/gyv045 (2015).

[25] Semprebon GM, Rivals F, Solounias N, Hulbert RC (2016). Paleodietary reconstruction of fossil horses from the Eocene through Pleistocene of North America. Palaeogeogr. Palaeoclimatol. Palaeoecol..

[26] Mihlbachler MC, Campbell D, Chen C, Ayoub M, Kaur P (2017). Microwear-mesowear congruence and mortality bias in rhinoceros mass-death assemblages. Paleobiology.

[27] Purnell MA, Darras LP (2016). Surface topography: metrology and properties. Surf. Topogr. Metrol. Prop..

[28] Purnell, M. A., Goodall, R. H., Thomson, S. & Matthews, C. J. D. Tooth microwear texture in odontocete whales: variation with tooth characteristics and implications for dietary analysis. *Biosurface Biotribology*, 10.1016/j.bsbt.2017.11.004 (2017).

[29] Scott RS (2006). Dental microwear texture analysis: technical considerations. J. Hum. Evol..

[30] Calandra I, Merceron G (2016). Dental microwear texture analysis in mammalian ecology. Mammal Rev..

[31] DeSantis LRG (2016). Dental microwear textures: reconstructing diets of fossil mammals. Surf. Topogr. Metrol. Prop..

[32] Ungar PS, Evans AR (2016). Exposing the past: surfce topography and texture of paleontolgoical and archeological remains. Surf. Topogr. Metrol. Prop..

[33] Arman SD (2016). Minimizing inter-microscope variability in dental microwear texture analysis. Surf. Topogr. Metrol. Prop..

[34] Ramdarshan, A. *et al*. Seeds, browse, and tooth wear: a sheep perspective. *Ecol*. *Evol*. 10.1002/ece3.2241 (2016). 10.1002/ece3.2241 PMC4983574 27547337

[35] Kaiser TM, Clauss M, Schulz-Kornas E (2016). A set of hypotheses on tribology of mammalian herbivore teeth. Surf. Topogr. Metrol. Prop..

[36] Blateyron, F. The areal field parameters. In *Characterisation of areal surface texture* 15–43 (Springer, 2013).

[37] Mihlbachler MC, Campbell D, Ayoub M, Chen C, Ghani I (2016). Comparative dental microwear of ruminant and perissodactyl molars: Implications for paleodietary analysis of rare and extinct ungulate clades. Paleobiology.

[38] Feir-Walsh BJ, Toothaker LE (1974). An empirical comparison of the ANOVA F-test, normal scores test and Kruskal-Wallis test under violation of assumptions. Educ. Psychol. Meas..

[39] Schmider E, Ziegler M, Danay E, Beyer L, Bühner M (2010). Is it really robust? Reinvestigating the robustness of ANOVA against violations of the normal distribution assumption. Methodology.

[40] Mihlbachler, M. C., Rusnack, F. & Brian L. Beatty. Experimental approaches to assess the effect of composition 1 of abrasives in the cause of. *J*. *Exp*. *Biol*. 10.1098/rsos.211549 PMC9174714 35706657

[41] Schulz, E. *et al*. Dietary abrasiveness in associated with variability of microwear and dental surface texture in rabbits. *PLoS ONE* **8** (2013). 10.1371/journal.pone.0056167 PMC3566079 23405263

[42] Schulz E, Calandra I, Kaiser T (2013). Feeding ecology and chewing mechanics in hoofed mammals: 3D tribology of enamel wear. Wear.

[43] Schulz E, Calandra I, Kaiser TM (2010). Applying tribology to the teeth of hoofed mammals. Scanning.

[44] Calandra, I., Schulz, E., Pinnow, M., Krohn, S. & Kaiser, T. Teasing apart the contributions of hard dietary items on 3D dental microtextures in primates. *J*. *Hum*. *Evol*. (2012). 10.1016/j.jhevol.2012.05.001 22705031

[45] Purnell MA, Crumpton N, Gill PG, Jones G, Rayfield EJ (2013). Within-guild dietary discrinimation from 3-D textrural analysis of tooth microwear in insectivorous mammals. J. Zool..

[46] Delezene LK (2013). Premolar microwear and tooth use in Australopithecus afarensis. J. Hum. Evol..

