# Supplementary material for: Surface Replication, Fidelity and Data Loss in Traditional Dental Microwear and Dental Microwear Texture Analysis
Source: Sci Rep. 2019 Feb 7;9:1595. doi: 10.1038/s41598-018-37682-5 (PMC6367376; doi:10.1038/s41598-018-37682-5)
Supplement: Supplementary file 1 — supplementary text, tables, figures [file 41598_2018_37682_MOESM1_ESM.docx]

SURFACE REPLICATION, FIDELITY AND DATA LOSS IN TRADITIONAL DENTAL MICROWEAR AND DENTAL MICROWEAR TEXTURE ANALYSIS

Matthew C. Mihlbachler^1, 2,*^

Melissa Foy^1^

Brian L. Beatty^1^

^1^ New York Institute of Technology College of Osteopathic Medicine, Old Westbury NY 11568

^2^ Division of Paleontology, American Museum of Natural History, New York, NY 10024

^*^Corresponding author ([mmihlbac@nyit.edu](mailto:mmihlbac@nyit.edu))

SUPPLIMENTARY INFORMATION

***Paired T test vs Wilcoxon signed-rank test***

These tests compare the average values of the ISO parameters and TM variables between the original surfaces and casts. The average values of 26 of 34 ISO parameters differed significantly between original and cast surfaces according to paired T-tests (Table S3). Nonparametric tests (Wilcoxon signed rank tests) found significant differences between originals and casts for 3 additional ISO parameters for a total of 29 out of 34 parameters. For TM variables, highly significant differences between original and cast surfaces were found for NS (narrow scratches), SP (small pits) and for LP (Wilcoxon signed-rank test only) (Table S4).

***ANOVA***

ANOVA of the raw data found significant differences between feeding trials in 23 out of 34 ISO parameters and in three of four TM variables (NS, LP, SP) (Tables S3, S4). The rank transformed ANOVA produced fewer significant results overall, with differences among only 15 of 34 ISO parameters.

In Tukey’s Post hoc pairwise comparisons made between the control group (P) to the remaining treatments, the rank transformed data found fewer significant differences (N=16) than analysis of the raw ISO data (N=18) (Table S5). The rank transformed analysis of cast data failed to find 6 of the significant differences located by the original data (type II errors). Rank transformed analysis of ISO cast data also produced two significant results where none were found in the original data (type I errors) involving feeding treatments Pde (ISO parameter Sv) and Dqs (ISO parameter Sdr). Anova of both raw and rank transformed data agree that the cast data were inferior to the original surfaces in finding differentiated aspects of surface texture among these feeding treatments. As hypothesized, type II errors in the cast results overwhelmingly predominated over type I errors although the rank transformed analysis resulting in more type I errors compared to the analysis of raw data. Tukey’s Post hoc pairwise comparisons of the ranked transformed TM data were very different than the analysis of raw data, with more type 1 errors (Table S6).

***Discriminant function analysis***

Discriminant function analysis of rank transformed data performed slightly better than the raw data in most instances with slightly higher rates of correct post hoc classification (Table S7). However, the relative performances of casts and original surfaces were similar. When TM or ISO data were analyzed separately, the casts produced slightly weaker discriminant functions than the original surfaces. Casts were 2.5-11.6 percentage points less successful than original data at post hoc classifications of specimens to their respective feeding treatments. Oddly, in the combined analysis of TM and ISO casts performance was only marginally inferior to the original data with a slightly lower Wilk’s Lambda and identical rate of correct post hoc classification (76.5%). Both raw and rank transformed data suggest that data derived from casts perform quite well in discriminant function analyses despite alterations to the data associated with casting wear surfaces.

Table S1. Means, and standard deviations of ISO parameters for tooth and cast surfaces for all feeding treatments combined. Abbreviations: MO (mean of original data); MC (mean of cast data); SO (standard deviation of original data); SC (standard deviation of cast data); RMS (relative mean shift); CVS (coefficient of variation shift).

| **parameter** | **MO** | **MC** | **SO** | **SC** | **RMS** | **CVS** |
| --- | --- | --- | --- | --- | --- | --- |
| **height parameters** | | | | | | |
| Sq | 0.56 | 0.59 | 0.18 | 0.20 | 0.16 | 0.00 |
| Ssk | -0.45 | -0.36 | 0.34 | 0.35 | 0.24 | -0.14 |
| Sku | 3.60 | 3.84 | 1.18 | 1.18 | 0.21 | -0.02 |
| Sp | 1.52 | 1.99 | 0.47 | 0.84 | 0.99 | 0.11 |
| Sv | 2.31 | 2.57 | 0.81 | 0.93 | 0.32 | 0.01 |
| Sz | 3.83 | 4.56 | 1.17 | 1.58 | 0.63 | 0.04 |
| Sa | 0.44 | 0.46 | 0.15 | 0.15 | 0.13 | 0.00 |
| **functional parameters** | | | | | | |
| Smr | 24.72 | 12.48 | 17.30 | 14.47 | -0.71 | 0.46 |
| Smc | 0.68 | 0.71 | 0.25 | 0.27 | 0.13 | 0.02 |
| Sxp | 1.26 | 1.29 | 0.45 | 0.45 | 0.07 | -0.01 |
| **spatial parameters** | | | | | | |
| Sal | 9.78 | 8.43 | 1.91 | 1.40 | -0.70 | -0.03 |
| Str | 0.41 | 0.45 | 0.17 | 0.17 | 0.21 | -0.01 |
| Std | 86.72 | 90.98 | 25.91 | 24.47 | 0.16 | -0.03 |
| **hybrid parameters** | | | | | | |
| Sdq | 0.27 | 0.34 | 0.09 | 0.13 | 0.79 | 0.06 |
| Sdr | 2.68 | 1.26 | 1.79 | 1.80 | 0.69 | 0.04 |
| **functional parameters (volume)** | | | | | | |
| Vm | 0.02 | 0.03 | 0.01 | 0.01 | 0.51 | 0.04 |
| Vv | 0.70 | 0.74 | 0.25 | 0.28 | 0.14 | 0.02 |
| Vmp | 0.02 | 0.03 | 0.01 | 0.01 | 0.46 | 0.04 |
| Vmc | 0.49 | 0.51 | 0.16 | 0.16 | 0.10 | -0.01 |
| Vvc | 0.63 | 0.66 | 0.23 | 0.26 | 0.14 | 0.02 |
| Vvv | 0.08 | 0.08 | 0.03 | 0.03 | 0.08 | -0.02 |
| **feature parameters** | | | | | | |
| Spd | 0.00 | 0.00 | 0.00 | 0.00 | 0.58 | 0.23 |
| Spc | 23.03 | 30.03 | 22.87 | 32.26 | 0.31 | 0.08 |
| S10z | 2.20 | 2.55 | 0.63 | 0.81 | 0.55 | 0.03 |
| S5p | 0.81 | 1.03 | 0.33 | 0.35 | 0.66 | -0.06 |
| S5v | 1.38 | 1.52 | 0.45 | 0.57 | 0.30 | 0.06 |
| Sda | 148.97 | 152.45 | 67.92 | 86.47 | 0.05 | 0.11 |
| Sha | 159.59 | 173.90 | 90.97 | 94.92 | 0.16 | -0.02 |
| Sdv | 3.23 | 3.95 | 6.22 | 5.08 | 0.12 | -0.64 |
| Shv | 4.24 | 5.48 | 4.04 | 5.15 | 0.31 | -0.01 |
| **other** | | | | | | |
| Iso | 40.81 | 44.5 | 17.22 | 18.14 | 0.21 | -0.01 |
| 1D | 81.77 | 85.58 | 26.51 | 29.15 | 0.14 | 0.02 |
| 2D | 85.47 | 82.41 | 33.65 | 32.69 | -0.09 | 0.00 |
| 3D | 79.87 | 86.13 | 37.19 | 40.09 | 0.17 | 0.00 |

Table S2. Means, and standard deviations of TM variables for tooth and cast surfaces for all feeding treatments combined. Abbreviations: MO (mean of original data); MC (mean of cast data); SO (standard deviation of original data); SC (standard deviation of cast data); RMS (relative mean shift); CVS (coefficient of variation shift).

| **variable** | **MO** | **MC** | **SO** | **SC** | **RMS** | **CVS** |
| --- | --- | --- | --- | --- | --- | --- |
| WS | 3.12 | 3.11 | 2.03 | 2.10 | 0.00 | 0.02 |
| NS | 17.13 | 28.68 | 8.15 | 8.76 | 1.42 | -0.17 |
| LP | 11.59 | 12.86 | 6.70 | 6.31 | 0.19 | -0.09 |
| SP | 61.14 | 85.08 | 21.25 | 20.82 | 1.13 | -0.10 |

Table S3. Parametric and nonparametric statistical results for tooth and cast surfaces with all feeding treatments combined. Statistical abbreviations: PCC (Pearson Correlation Coefficients), Paired T (Related samples paired T tests); Wilcoxon SRT (Wilcoxon signed rank test); ANOVA (analysis of variance) and RT ANOVA (analysis of variance on rank transformed data). Dark cells contain significant results(P≤0.05). See Table 1 for abbreviations.

| **parameter name** | **PCC** | **Paired T**  **P** | **Wilcoxon SRT**  **P** | **Anova P (teeth)** | **RT Anova P**  **(teeth)** | **Anova**  **P**  **(casts)** | **RT Anova P (casts)** |
| --- | --- | --- | --- | --- | --- | --- | --- |
| **Height parameters** | | | | | | | |
| Sq | 0.919 | < 0.001 | < 0.001 | < 0.001 | = 0.001 | < 0.001 | < 0.001 |
| Ssk | 0.492 | = 0.011 | = 0.005 | = 0.142 | = 0.102 | = 0.088 | = 0.066 |
| Sku | 0.721 | = 0.005 | = 0.006 | = 0.031 | = 0.229 | = 0.160 | = 0.450 |
| Sp | 0.653 | < 0.001 | < 0.001 | = 0.013 | = 0.062 | = 0.002 | = 0.009 |
| Sv | 0.817 | < 0.001 | < 0.001 | = 0.006 | = 0.001 | = 0.005 | < 0.001 |
| Sz | 0.821 | < 0.001 | < 0.001 | = 0.005 | = 0.004 | = 0.002 | < 0.001 |
| Sa | 0.918 | = 0.001 | < 0.001 | < 0.001 | < 0.001 | < 0.001 | < 0.001 |
| **Functional parameters** | | | | | | | |
| Smr | 0.462 | < 0.001 | < 0.001 | = 0.013 | = 0.171 | = 0.026 | = 0.046 |
| Smc | 0.917 | = 0.001 | < 0.001 | < 0.001 | = 0.002 | < 0.001 | < 0.001 |
| Sxp | 0.899 | = 0.082 | = 0.035 | = 0.002 | < 0.001 | = 0.004 | < 0.001 |
| **Spatial parameters** | | | | | | | |
| Sal | 0.697 | < 0.001 | < 0.001 | = 0.163 | = 0.248 | = 0.330 | = 0.258 |
| Str | 0.635 | = 0.009 | = 0.019 | = 0.417 | = 0.827 | = 0.842 | = 0.867 |
| Std | 0.526 | = 0.051 | = 0.019 | = 0.543 | = 0.240 | = 0.319 | = 0.146 |
| **Hybrid parameters** | | | | | | | |
| Sdq | 0.730 | < 0.001 | < 0.001 | < 0.001 | < 0.001 | < 0.001 | < 0.001 |
| Sdr | 0.732 | < 0.001 | < 0.001 | < 0.001 | < 0.001 | < 0.001 | < 0.001 |
| **Functional parameters (volume)** | | | | | | | |
| Vm | 0.692 | < 0.001 | < 0.001 | = 0.018 | = 0.203 | = 0.031 | = 0.090 |
| Vv | 0.918 | < 0.001 | < 0.001 | < 0.001 | < 0.001 | < 0.001 | < 0.001 |
| Vmp | 0.590 | < 0.001 | < 0.001 | = 0.005 | == 0.203 | = 0.009 | = 0.090 |
| Vmc | 0.903 | = 0.012 | = 0.001 | < 0.001 | < 0.001 | < 0.001 | < 0.001 |
| Vvc | 0.913 | < 0.001 | < 0.001 | < 0.001 | = 0.003 | < 0.001 | < 0.001 |
| Vvv | 0.887 | < 0.001 | = 0.026 | = 0.015 | < 0.001 | = 0.018 | < 0.001 |
| **Feature parameters** | | | | | | | |
| Spd | 0.469 | < 0.001 | < 0.001 | = 0.010 | = 0.622 | = 0.008 | = 0.293 |
| Spc | 0.709 | = 0.001 | = 0.004 | < 0.001 | < 0.001 | < 0.001 | < 0.001 |
| S10z | 0.763 | < 0.001 | < 0.001 | = 0.003 | = 0.002 | = 0.003 | = 0.002 |
| S5p | 0.497 | < 0.001 | < 0.001 | = 0.003 | = 0.325 | = 0.001 | = 0.622 |
| S5v | 0.697 | < 0.001 | < 0.001 | = 0.011 | < 0.001 | = 0.005 | < 0.001 |
| Sda | 0.211 | = 0.663 | = 0.779 | = 0.453 | = 0.222 | = 0.265 | = 0.209 |
| Sha | 0.487 | = 0.093 | = 0.236 | = 0.048 | = 0.193 | = 0.089 | = 0.237 |
| Sdv | 0.194 | = 0.260 | < 0.001 | = 0.594 | = 0.138 | = 0.101 | = 0.519 |
| Shv | 0.377 | = 0.009 | = 0.002 | = 0.155 | = 0.286 | = 0.395 | = 0.665 |
| **Other** | | | | | | | |
| Isotropy | 0.635 | = 0.009 | = 0.019 | = 0.417 | = 0.827 | = 0.842 | = 0.867 |
| First Direction | 0.615 | = 0.091 | = 0.135 | = 0.639 | = 0.621 | = 0.373 | = 0.873 |
| Second Direction | 0.086 | = 0.456 | = 0.575 | = 0.996 | = 0.674 | = 0.998 | = 0.931 |
| Third Direction | 0.319 | = 0.118 | = 0.151 | 0.418 | = 0.666 | = 0.359 | = 0.762 |

Table S4. Statistical results for traditional microwear variables of tooth and cast surfaces with all feeding treatments combined. PCC (Pearson Correlation Coefficients), Paired T (Related samples paired T tests) and Wilcoxon SRT (signed rank test) are results that compare teeth to casts. Anova and RT (rank transformed) ANOVA independently test the ability of teeth and casts to find differences between the feeding treatments. Dark cells contain significant results(P<0.05).

| **variable name** | **PCC** | **Paired T**  **P** | **Wilcoxon SRT**  **P** | **Anova P (teeth)** | **RT Anova (teeth)**  **P** | **Anova**  **(casts)**  **P** | **RT Anova (casts)**  **P** |
| --- | --- | --- | --- | --- | --- | --- | --- |
| wide scratches (WS) | = 0.435 | = 0.967 | = 0.833 | = 0.707 | = 0.096 | = 0.631 | = 0.353 |
| narrow scratches (NS) | = 0.455 | < 0.001 | < 0.001 | < 0.001 | = 0.003 | < 0.001 | < 0.001 |
| large pits (LP) | = 0.411 | = 0.052 | = 0.007 | = 0.003 | < 0.001 | < 0.001 | < 0.001 |
| small pits (SP) | = 0.340 | < 0.001 | < 0.001 | = 0.001 | = 0.006 | < 0.001 | = 0.001 |

Table S5. Tukey’s test P-values for pairwise comparisons of ISO parameters based on ANOVA of rank transformed data. The value 0.000 indicates P < 0.001, all other values are equal to P as reported by SPSS. Dark cells contain significant results (P ≤ 0.05). Abbreviations are as in tables 1 and 3. ISO parameters with no significant results are not shown.

| **Tooth surfaces** | | | | | | | | | | | | | | | | |
| --- | --- | --- | --- | --- | --- | --- | --- | --- | --- | --- | --- | --- | --- | --- | --- | --- |
| **Pellet vs** | Sq | Sp | Sv | Sa | Smc | Sxp | Sdq | Sdr | Vv | Vmp | Vmc | Vvc | Spd | Spc | S10z | S5p |
| **D** | 0.322 | 0.995 | 1.000 | 0.198 | 0.123 | 0.431 | 1.000 | 0.956 | 0.143 | 0.999 | 0.158 | 0.128 | 0.972 | 0.666 | 0.923 | 0.799 |
| **Pcc** | 0.380 | 0.999 | 0.999 | 0.195 | 0.094 | 0.950 | 0.969 | 0.859 | 0.097 | 0.991 | 0.129 | 0.059 | 0.997 | 1.000 | 0.239 | 0.642 |
| **Pde** | 1.000 | 0.951 | 0.197 | 0.999 | 0.976 | 1.000 | 0.002 | 0.006 | 0.982 | 1.000 | 0.969 | 0.949 | 0.903 | 0.048 | 0.998 | 1.000 |
| **Pqs** | 0.400 | 1.000 | 1.000 | 0.207 | 0.217 | 0.558 | 0.995 | 0.989 | 0.233 | 0.973 | 0.133 | 0.203 | 0.997 | 0.958 | 0.999 | 0.921 |
| **Dcc** | 0.544 | 0.971 | 1.000 | 0.396 | 0.329 | 0.722 | 0.997 | 1.000 | 0.335 | 0.671 | 0.325 | 0.282 | 0.066 | 0.734 | 0.918 | 0.998 |
| **Dde** | 0.562 | 0.608 | 0.711 | 0.245 | 0.141 | 0.988 | 0.026 | 0.050 | 0.146 | 0.776 | 0.168 | 0.070 | 0.025 | 0.504 | 0.996 | 0.868 |
| **Dqs** | 0.000 | 0.023 | 0.913 | 0.000 | 0.000 | 0.007 | 0.228 | 0.055 | 0.000 | 0.020 | 0.000 | 0.000 | 0.249 | 0.192 | 0.023 | 0.011 |
|  | | | | | | | | | | | | | | | | |
| **Cast surfaces** | | | | | | | | | | | | | | | | |
| **Pellet vs** | Sq | Sp | Sv | Sa | Smc | Sxp | Sdq | Sdr | Vv | Vmp | Vmc | Vvc | Spd | Spc | S10z | S5p |
| **D** | 0.487 | 1.000 | 1.000 | 0.402 | 0.479 | 0.319 | 0.994 | 0.854 | 0.521 | 1.000 | 0.329 | 0.631 | 1.000 | 1.000 | 1.000 | 1.000 |
| **Pcc** | 0.792 | 1.000 | 0.993 | 0.647 | 0.517 | 0.992 | 0.916 | 0.775 | 0.591 | 0.998 | 0.539 | 0.569 | 1.000 | 1.000 | 1.000 | 1.000 |
| **Pde** | 0.891 | 0.974 | 0.020 | 0.974 | 0.990 | 0.805 | 0.001 | 0.006 | 0.983 | 0.943 | 1.000 | 0.992 | 0.989 | 0.018 | 0.164 | 0.908 |
| **Pqs** | 0.903 | 0.907 | 1.000 | 0.784 | 0.933 | 0.821 | 1.000 | 1.000 | 0.954 | 1.000 | 0.596 | 0.966 | 0.999 | 1.000 | 1.000 | 1.000 |
| **Dcc** | 0.335 | 0.768 | 1.000 | 0.290 | 0.361 | 0.333 | 1.000 | 0.988 | 0.416 | 0.913 | 0.212 | 0.534 | 0.808 | 1.000 | 0.832 | 1.000 |
| **Dde** | 0.962 | 0.999 | 0.653 | 0.834 | 0.886 | 1.000 | 0.195 | 0.358 | 0.905 | 0.999 | 0.656 | 0.867 | 0.585 | 0.245 | 0.981 | 0.994 |
| **Dqs** | 0.008 | 0.091 | 0.979 | 0.002 | 0.002 | 0.041 | 0.056 | 0.039 | 0.002 | 0.415 | 0.000 | 0.002 | 0.791 | 0.0550 | 0.707 | 1.000 |

Table S6. Tukey’s test P-values for pairwise comparisons of TM variables of ISO parameters based on ANOVA of rank transformed data. The value 0.000 indicates P < 0.001, all other values are equal to P as reported by SPSS. Dark cells contain significant results (P ≤ 0.05).. Abbreviations are as in tables 2 and 3.

| **Tooth surfaces** | | | | |
| --- | --- | --- | --- | --- |
| **Pellet vs** | WS | NS | LP | SP |
| **D** | 1.000 | 0.725 | 0.998 | 0.981 |
| **Pcc** | 1.000 | 0.798 | 0.998 | 0.987 |
| **Pde** | 1.000 | 0.688 | 0.658 | 0.348 |
| **Pqs** | 1.000 | 0.544 | 1.000 | 0.929 |
| **Dcc** | 1.000 | 1.000 | 0.999 | 0.984 |
| **Dde** | 0.983 | 0.999 | 0.001 | 0.032 |
| **Dqs** | 0.945 | 0.000 | 1.000 | 1.000 |
|  | | | | |
| **Cast surfaces** | | | | |
| **Pellet vs** | WS | NS | LP | SP |
| **D** | 1.000 | 0.854 | 0.998 | 1.000 |
| **Pde** | 0.858 | 0.535 | 0.002 | 0.037 |
| **Pcc** | 0.979 | 0.990 | 0.838 | 0.865 |
| **Pqs** | 1.000 | 0.002 | 1.000 | 1.000 |
| **Dcc** | 1.000 | 0.999 | 0.072 | 0.280 |
| **Dde** | 0.953 | 0.869 | 0.005 | 0.249 |
| **Dqs** | 1.000 | 1.000 | 0.978 | 0.977 |

Table S7. Results of discriminant function analyses based on rank transformed data from original tooth surfaces and cast surfaces.

| **Tooth surfaces** | | | | | | | |
| --- | --- | --- | --- | --- | --- | --- | --- |
|  | Chi-Squared | Sig. (P) | Wilks' Lambda | % correctly classified | % var F1 | % var F2 | % var F3 |
| TM | 120.362 | P<0.001 | 0.345 | 43.3 | 52.7 | 40.3 | 6.0 |
| ISO | 319.818 | P<0.001 | 0.043 | 72.4 | 34.5 | 20.7 | 16.1 |
| ISO + TM | 372.282 | P<0.001 | 0.021 | 76.5 | 30.8 | 29.4 | 15.2 |
|  | | | | | | | |
| **Cast Surfaces** | | | | | | | |
|  | Chi-Squared | Sig. (P) | Wilks' Lambda | % correctly classified | % var F1 | % var F2 | % var F3 |
| TM | 82.211 | P<0.001 | 0.466 | 31.7 | 61.9 | 33.9 | 3.5 |
| ISO | 294.514 | P=0.006 | 0.057 | 69.9 | 38.3 | 20.3 | 14.4 |
| ISO + TM | 35.979 | P<0.001 | 0.025 | 76.5 | 34.4 | 21.7 | 15.3 |

Table S8. Canonical loadings for seven discriminant functions (DF1–DF7) of TM and ISO variables based on the total evidence analysis of original teeth.

|  | DF 1 | DF 2 | DF 3 | DF 4 | DF 5 | DF 6 | DF 7 |
| --- | --- | --- | --- | --- | --- | --- | --- |
| % variance explained | 38.7 | 23.7 | 12.4 | 8.7 | 6.5 | 5.3 | 4.8 |
|  | | | | | | | |
| Sq | -0.333 | 0.039 | 0.288 | 0.079 | -0.033 | -0.136 | 0.021 |
| Ssk | -0.095 | -0.280 | 0.115 | 0.203 | 0.019 | 0.245 | 0.053 |
| Sku | 0.174 | 0.183 | -0.001 | -0.328 | -0.033 | -0.209 | 0.221 |
| Sp | -0.214 | -0.031 | 0.300 | -0.038 | -0.014 | -0.280 | 0.167 |
| Sv | -0.148 | 0.248 | 0.305 | -0.060 | 0.024 | -0.272 | 0.152 |
| Sz | -0.190 | 0.157 | 0.334 | -0.057 | 0.011 | -0.303 | 0.174 |
| Sa | -0.343 | 0.029 | 0.305 | 0.104 | -0.036 | -0.107 | 0.003 |
| Smr | 0.163 | 0.078 | -0.276 | 0.151 | 0.081 | 0.437 | -0.126 |
| Smc | -0.349 | 0.008 | 0.308 | 0.094 | -0.038 | -0.048 | 0.038 |
| Sxp | -0.278 | 0.099 | 0.175 | 0.047 | -0.014 | -0.228 | -0.048 |
| Sal | -0.133 | -0.086 | -0.166 | -0.136 | 0.332 | -0.088 | 0.129 |
| Str | 0.023 | 0.097 | -0.189 | 0.102 | -0.045 | -0.355 | 0.365 |
| Std | -0.046 | -0.009 | 0.070 | -0.076 | 0.099 | 0.359 | -0.050 |
| Sdq | -0.201 | 0.467 | 0.450 | -0.065 | -0.069 | -0.307 | 0.040 |
| Sdr | -0.258 | 0.417 | 0.390 | -0.098 | -0.101 | -0.280 | 0.052 |
| Vm | -0.239 | -0.049 | 0.233 | 0.029 | -0.010 | -0.138 | 0.232 |
| Vv | -0.348 | 0.006 | 0.309 | 0.093 | -0.038 | -0.051 | 0.044 |
| Vmp | -0.284 | -0.023 | 0.271 | 0.045 | 0.048 | -0.159 | 0.191 |
| Vmc | -0.356 | 0.026 | 0.299 | 0.127 | -0.038 | -0.059 | -0.030 |
| Vvc | -0.347 | -0.004 | 0.318 | 0.096 | -0.040 | -0.024 | 0.052 |
| Vvv | -0.259 | 0.093 | 0.144 | 0.046 | -0.010 | -0.258 | -0.034 |
| Spd | 0.193 | 0.253 | 0.032 | -0.175 | 0.008 | 0.158 | -0.200 |
| Spc | -0.262 | 0.264 | 0.469 | -0.061 | 0.343 | -0.175 | -0.008 |
| S10z | -0.226 | 0.106 | 0.331 | 0.042 | -0.118 | -0.091 | 0.179 |
| S5p | -0.266 | -0.055 | 0.222 | -0.143 | -0.257 | 0.159 | 0.270 |
| S5v | -0.127 | 0.193 | 0.309 | 0.166 | 0.021 | -0.248 | 0.056 |
| Sda | -0.138 | -0.095 | -0.095 | 0.060 | -0.013 | 0.024 | 0.164 |
| Sha | -0.097 | -0.137 | -0.297 | 0.250 | 0.220 | 0.093 | 0.165 |
| Sdv | -0.142 | -0.057 | 0.056 | -0.110 | -0.096 | -0.005 | 0.044 |
| Shv | -0.212 | 0.048 | -0.078 | 0.133 | 0.139 | 0.027 | 0.113 |
| Isotropy | 0.023 | 0.097 | -0.189 | 0.102 | -0.045 | -0.355 | 0.365 |
| First Direction | 0.043 | -0.126 | -0.106 | -0.037 | 0.016 | 0.163 | -0.229 |
| Second Direction | 0.036 | -0.037 | 0.064 | -0.039 | -0.008 | 0.018 | 0.072 |
| Third Direction | 0.128 | -0.003 | 0.009 | 0.215 | 0.003 | 0.208 | 0.330 |
| WS | 0.004 | 0.133 | -0.001 | 0.155 | 0.049 | 0.166 | -0.163 |
| NS | 0.580 | 0.131 | 0.093 | 0.026 | -0.021 | -0.134 | 0.057 |
| LP | -0.095 | 0.397 | -0.145 | 0.057 | -0.106 | 0.035 | -0.131 |
| SP | -0.089 | 0.419 | 0.308 | 0.034 | -0.044 | -0.078 | -0.048 |

Table S9. Canonical loadings for seven discriminant functions (DF1–DF7) of TM and ISO variables based on the total evidence analysis of casts.

|  | DF 1 | DF 2 | DF 3 | DF 4 | DF 5 | DF 6 | DF 7 |
| --- | --- | --- | --- | --- | --- | --- | --- |
| % variance explained | 38.6 | 22 | 13.3 | 11.5 | 7.7 | 3.8 | 3 |
|  | | | | | | | |
| Sq | 0.234 | 0.351 | -0.037 | -0.097 | -0.273 | -0.178 | 0.181 |
| Ssk | -0.124 | 0.176 | 0.013 | 0.321 | 0.181 | -0.129 | 0.201 |
| Sku | 0.082 | -0.213 | 0.050 | -0.230 | -0.019 | -0.074 | -0.229 |
| Sp | 0.129 | 0.302 | 0.049 | -0.103 | -0.026 | -0.143 | 0.220 |
| Sv | 0.326 | 0.170 | 0.035 | -0.219 | -0.186 | -0.117 | 0.082 |
| Sz | 0.258 | 0.262 | 0.047 | -0.183 | -0.122 | -0.145 | 0.166 |
| Sa | 0.234 | 0.369 | -0.022 | -0.071 | -0.280 | -0.163 | 0.212 |
| Smr | -0.085 | -0.205 | -0.115 | 0.226 | 0.042 | -0.121 | -0.182 |
| Smc | 0.229 | 0.343 | -0.016 | -0.020 | -0.217 | -0.220 | 0.126 |
| Sxp | 0.236 | 0.349 | -0.097 | -0.212 | -0.276 | -0.065 | 0.145 |
| Sal | -0.026 | 0.239 | -0.005 | -0.294 | 0.019 | -0.308 | 0.036 |
| Str | 0.047 | -0.075 | -0.071 | -0.228 | 0.056 | 0.036 | -0.231 |
| Std | -0.095 | 0.002 | 0.247 | 0.176 | -0.206 | -0.017 | -0.373 |
| Sdq | 0.490 | 0.273 | 0.009 | -0.180 | -0.266 | -0.127 | 0.142 |
| Sdr | 0.445 | 0.303 | 0.013 | -0.160 | -0.306 | -0.132 | 0.104 |
| Vm | 0.102 | 0.253 | -0.076 | -0.036 | -0.152 | -0.283 | 0.161 |
| Vv | 0.227 | 0.344 | -0.018 | -0.021 | -0.217 | -0.225 | 0.129 |
| Vmp | 0.102 | 0.253 | -0.076 | -0.036 | -0.152 | -0.283 | 0.161 |
| Vmc | 0.234 | 0.383 | -0.007 | -0.063 | -0.304 | -0.135 | 0.288 |
| Vvc | 0.221 | 0.340 | -0.012 | 0.005 | -0.205 | -0.240 | 0.126 |
| Vvv | 0.233 | 0.311 | -0.070 | -0.258 | -0.288 | -0.030 | 0.132 |
| Spd | 0.147 | -0.118 | -0.020 | 0.070 | 0.043 | 0.021 | -0.062 |
| Spc | 0.355 | 0.289 | -0.029 | 0.021 | -0.060 | -0.021 | 0.043 |
| S10z | 0.246 | 0.304 | 0.091 | -0.219 | -0.004 | -0.237 | 0.068 |
| S5p | 0.091 | 0.185 | 0.069 | -0.035 | -0.064 | -0.291 | 0.306 |
| S5v | 0.298 | 0.317 | 0.086 | -0.296 | 0.038 | -0.146 | -0.111 |
| Sda | -0.157 | -0.080 | -0.304 | 0.037 | -0.077 | -0.001 | -0.209 |
| Sha | -0.088 | 0.197 | 0.242 | 0.081 | -0.008 | 0.130 | 0.264 |
| Sdv | 0.040 | -0.025 | -0.355 | -0.062 | -0.201 | -0.073 | -0.336 |
| Shv | 0.091 | 0.191 | 0.180 | 0.082 | 0.032 | 0.320 | 0.000 |
| Isotropy | 0.047 | -0.075 | -0.071 | -0.228 | 0.056 | 0.036 | -0.231 |
| First Direction | -0.149 | 0.004 | -0.026 | 0.171 | 0.088 | -0.073 | -0.046 |
| Second Direction | -0.044 | -0.006 | 0.012 | 0.272 | -0.154 | -0.104 | 0.131 |
| Third Direction | 0.118 | -0.136 | -0.036 | 0.073 | 0.035 | -0.038 | -0.058 |
| WS | 0.192 | -0.140 | 0.176 | 0.034 | -0.252 | 0.099 | 0.058 |
| NS | 0.002 | -0.440 | 0.101 | -0.142 | -0.105 | 0.200 | 0.075 |
| LP | 0.428 | 0.039 | -0.218 | -0.083 | 0.125 | 0.137 | 0.111 |
| SP | 0.275 | 0.064 | -0.324 | -0.102 | 0.087 | -0.072 | 0.212 |


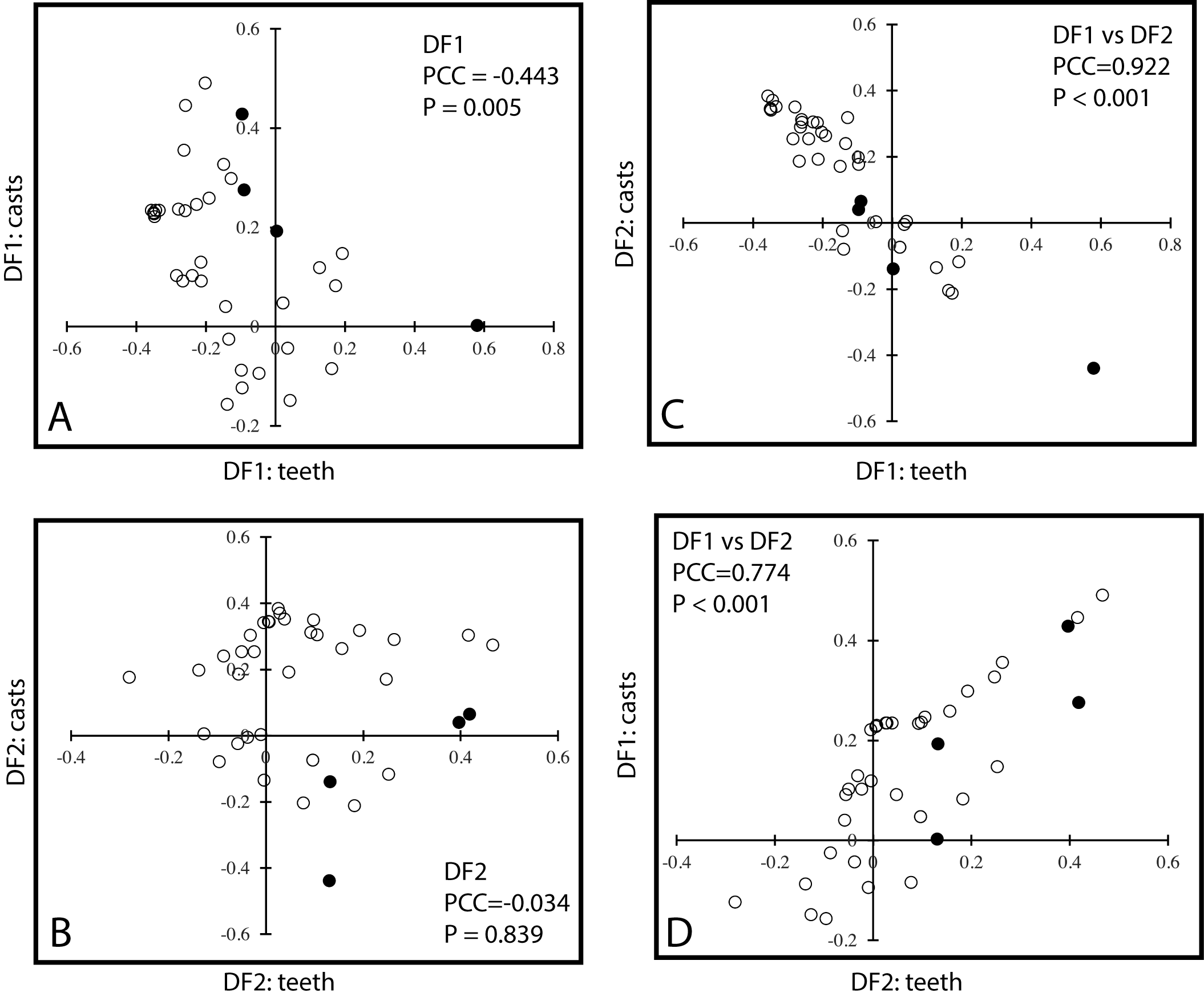
Figure S1. Correlations of canonical loadings for discriminant functions derived from the total evidence analysis of original surfaces and cast surfaces. Open circles are ISO parameters, filled circles are TM variables.


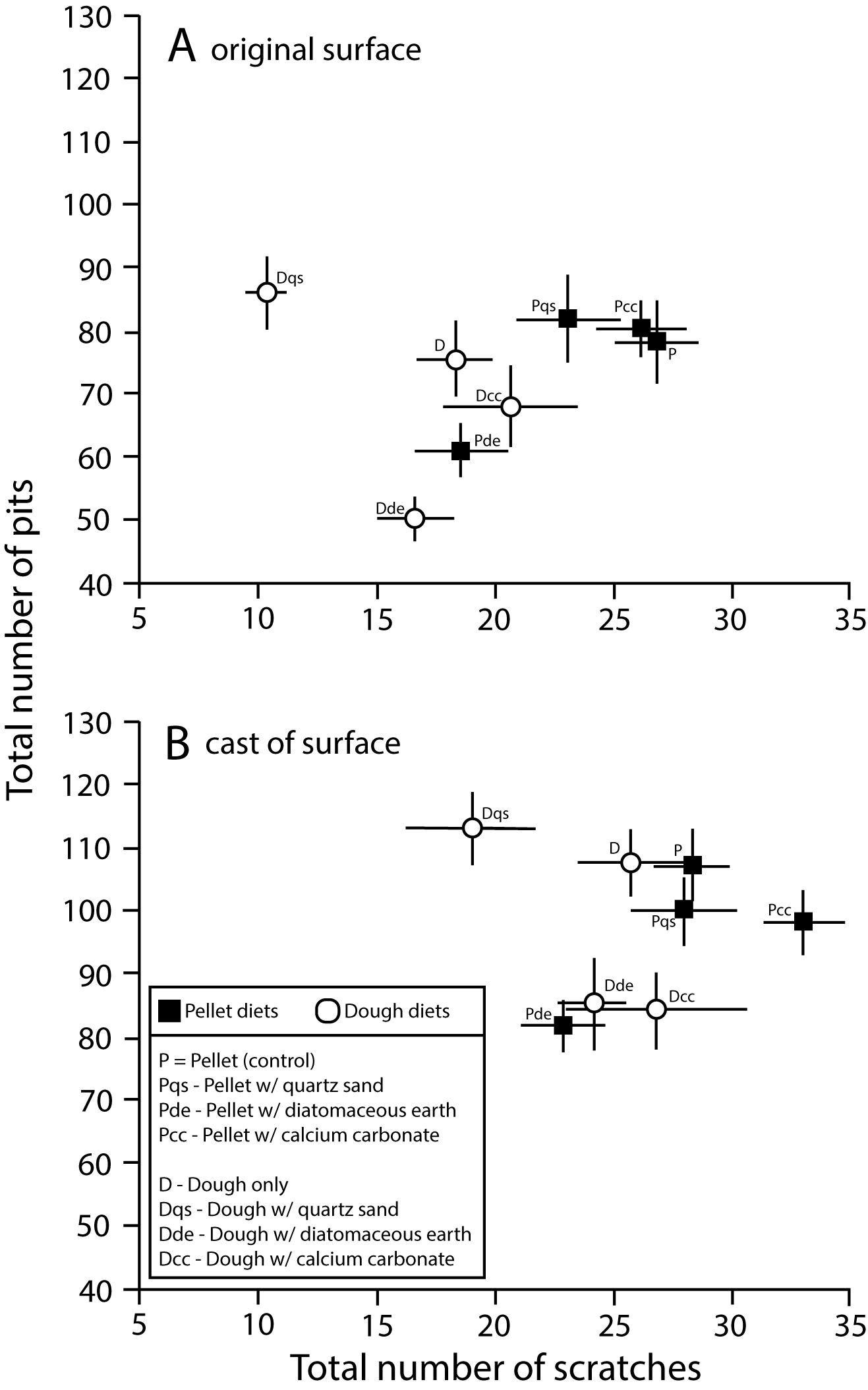


Figure S2. Means and standard errors of TM variables, total number of scratches and total number of pits for original tooth surfaces (A) and casts (B).
